# Local adaptations of Mediterranean sheep and goats through an integrative approach

**DOI:** 10.1038/s41598-021-00682-z

**Published:** 2021-11-01

**Authors:** Bruno Serranito, Marco Cavalazzi, Pablo Vidal, Dominique Taurisson-Mouret, Elena Ciani, Marie Bal, Eric Rouvellac, Bertrand Servin, Carole Moreno-Romieux, Gwenola Tosser-Klopp, Stephen J. G. Hall, Johannes A. Lenstra, François Pompanon, Badr Benjelloun, Anne Da Silva

**Affiliations:** 1 INRA, EA7500, USC1061 GAMAA, Univ. Limoges, 87000 Limoges, France; 2 CRESCO, Museum National d’Histoire Naturelle (MNHN), 35800 Dinard, France; 3 GEOLAB, UMR 6042, Univ. Limoges, Limoges, France; 4 Universidad Catolica de Valencia, Valencia, Spain; 5 CNRS, UMR 5815, Dynamiques du droit, Université de Montpellier, Montpellier, France; 6 Department of Biosciences, Biotechnologies and Biopharmaceutics, University of Bari, Bari, Italy; 7 GenPhySE, INRAE, ENVT, Université de Toulouse, 31326 Castanet-Tolosan, France; 8 Estonian University of Life Sciences, Kreutzwaldi 5, 51014 Tartu, Estonia; 9 Faculty of Veterinary Medicine, Utrecht University, Yalelaan 104, 3584 CM Utrecht, The Netherlands; 10 Univ. Grenoble Alpes, Univ. Savoie Mont Blanc, CNRS, LECA, F-38000 Grenoble, France; 11 National Institute of Agronomic Research (INRA), Regional Centre of Agronomic Research, Beni-Mellal, Morocco

**Keywords:** Population genetics, Animal breeding, Ecological genetics, Molecular biology

## Abstract

Small ruminants are suited to a wide variety of habitats and thus represent promising study models for identifying genes underlying adaptations. Here, we considered local Mediterranean breeds of goats (n = 17) and sheep (n = 25) from Italy, France and Spain. Based on historical archives, we selected the breeds potentially most linked to a territory and defined their original cradle (i.e., the geographical area in which the breed has emerged), including transhumant pastoral areas. We then used the programs PCAdapt and LFMM to identify signatures of artificial and environmental selection. Considering cradles instead of current GPS coordinates resulted in a greater number of signatures identified by the LFMM analysis. The results, combined with a systematic literature review, revealed a set of genes with potentially key adaptive roles in relation to the gradient of aridity and altitude. Some of these genes have been previously implicated in lipid metabolism (SUCLG2, BMP2), hypoxia stress/lung function (BMPR2), seasonal patterns (SOX2, DPH6) or neuronal function (TRPC4, TRPC6). Selection signatures involving the PCDH9 and KLH1 genes, as well as NBEA/NBEAL1, were identified in both species and thus could play an important adaptive role.

## Introduction

Sheep and goats were among the first mammalian livestock species to be domesticated, in a process that started at ca. 10,000–9500 B.P. in Mesopotamia^1^. The subsequent expansion across the Mediterranean Basin via maritime transport included different waves of colonization. The remains testifying to these founding periods along the northern coasts of the western Mediterranean are dated from 8100 to 7700 B.P.^2^.

Waves of dispersal, which have dispatched small groups of individuals (founder effect) to very diverse habitats, are the starting point of the process that led to the emergence of breeds^3^. Traditional pastoralism, characterized by limited human intervention (i.e., “soft” artificial selection^4^), and in conjunction with a strong influence of natural selection, led over the millennia to populations genetically differentiated and adapted to various agro-climatic conditions^5^. Sheep and goats provide an interesting model for deepening our knowledge of adaptive mechanisms under extreme environmental conditions.

In industrialized countries, artificial breeding has intensified over the past 200 years^4^, focusing mainly on agronomic traits related to milk, meat production and fiber quality. Many breeds have experienced negative impacts on their ability to adapt, their level of hardiness and sometimes their fitness by crossbreeding and/or intensive artificial selection^4, 6^. In contrast, the so-called local breeds have remained at least partially, connected to their original environments. We suggest it is among these local breeds that selection signatures related to environmental adaptation will be most detectable. For example, studies on local sheep in Ethiopia^7^ and Tibet^8, 9^ have identified several genes involved in adaptation to thermal stress and hypoxia. Kim et al.^10^, considering both local goats and sheep of hot and arid environment, have highlighted genes implicated in the adaptation to thermal stress.

This study aimed at identifying the genomic bases of adaptation in local breeds of goats and sheep distributed along the Mediterranean arc, in Italy, the south of France and Spain. The area under consideration harbours contrasting environments, such as high altitude areas (Alps, Pyrenees, Corsica), coastal areas, wetlands and arid areas, with closely related sheep and goat breeds^11–13^. All these features provide an ideal situation for the identification of adaptation signatures.

We hypothesize that selection signatures relevant to environmental adaptation, will be more reliably identified by reference to the environmental conditions of the breed’s cradle of origin, rather than to those of the locations where the animals were actually sampled. Indeed, breeds of domestic animals are at the interface between anthropized and natural environments; their evolutionary trajectory is thus determined by both the environment and human societies. First, (i) sheep and goat breeds have originally emerged within socially structured human groups, sometimes referred to as clans or tribes, occupying a well-defined territory, and within which herds were transmitted from generation to generation^14, 15^. In industrialized countries, the traditional links between territory, social group and breeds has become distorted. Thus, herds of a given breed can be found outside of its original cradle. Hence, the search for a selection signature may be optimized by considering the environmental conditions of the breed’s cradle, where the genomes have been shaped over time, rather than the current location of herds. Moreover, (ii) we should consider that traditional pastoralism was largely based on transhumance. During these periods, the animals are kept outdoors, most often in mountain altitude summer pastures, such that the strength of natural selection is particularly pronounced. Even if transhumance practices are increasingly abandoned^16, 17^, they have fundamentally shaped the genetic adaptations of sheep and goats.

The objective of this study was to investigate the regions of the genome underlying environmental adaptations, i.e. selection signatures linked to environmental variables. Selection signatures are identified as genomic areas comprising a number of neighboring markers under selection. We used the PCAdapt^18^ and LFMM^19^ methods. PCAdapt uses principal component analysis to describe population structure and identifies candidate markers as outliers in terms of inferred population structure. The LFMM approach searches for significant associations with environmental factors while controlling for neutral population structure. These approaches were coupled with a thorough analysis of the literature to identify the signatures including genes most likely related to environment adaptations. Moreover, we used datasets that have been previously investigated, in order to assess if the use of historical and anthropological dimensions of breeds improves the detection of selection signatures. We reasoned that (i) the inclusion of authentic local breeds in the analysis will limit the background noise caused by breeds for which the link to the environment is weak or has eroded, for instance by crossbreeding or migration; and (ii) targeting the geographical area in which populations have evolved rather than the present distribution will optimize the identification of adaptive genomic regions. Next, the proposed approach was implemented to search for selection signatures underlying local adaptation. We attached particular importance to the selection signatures involving homologous genomic regions in sheep and goats.

## Results

### Breed analyses

**Figure 1:**
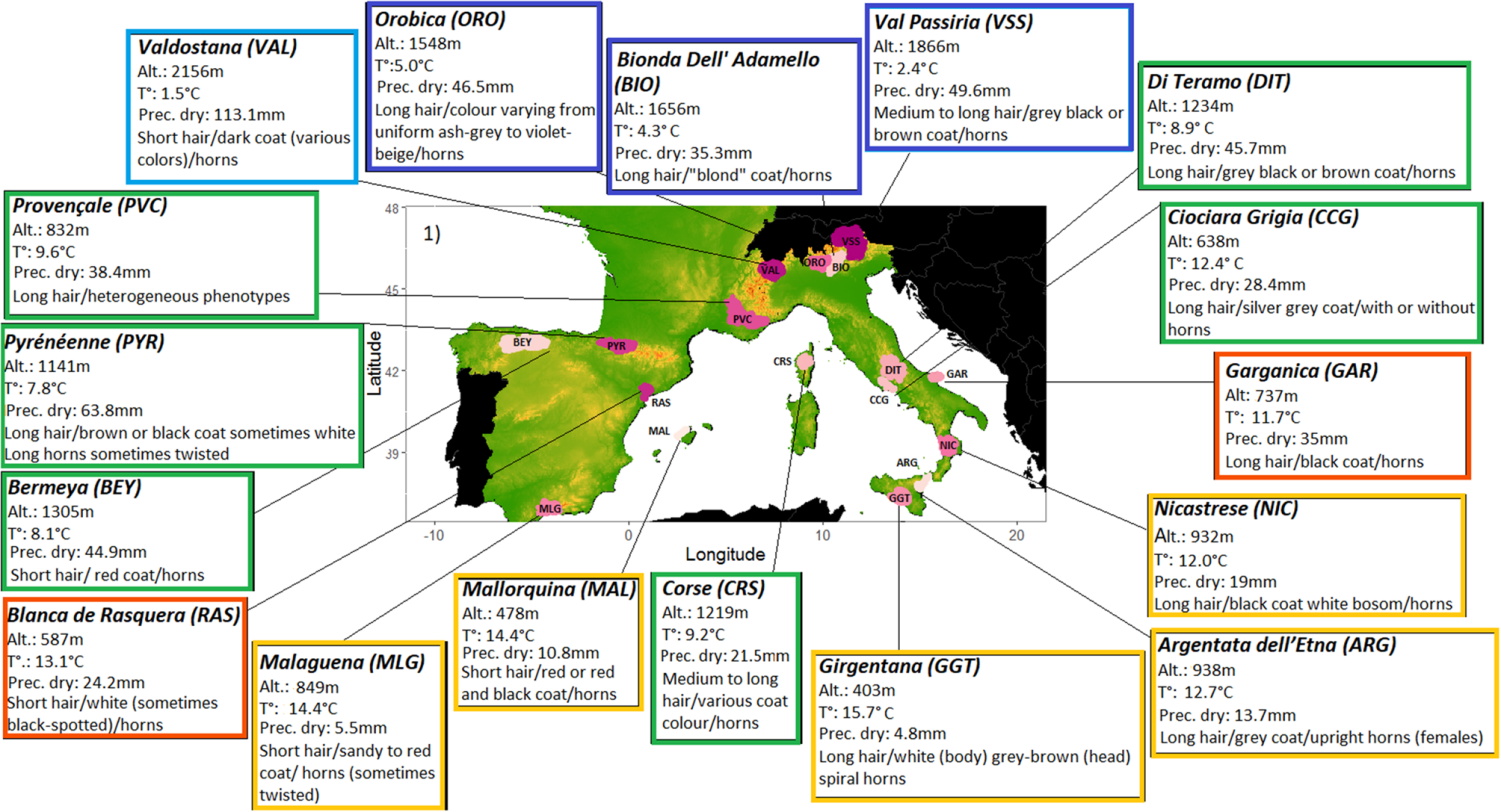
Description of goat cradles as a function of environmental variables: synthetic descriptions of cradles and some characteristic breed phenotypic traits. The breed labels are color coded according to the clustering ranking defined by the PCA/HCPC analysis shown in Fig. 4.

**Figure 2:**
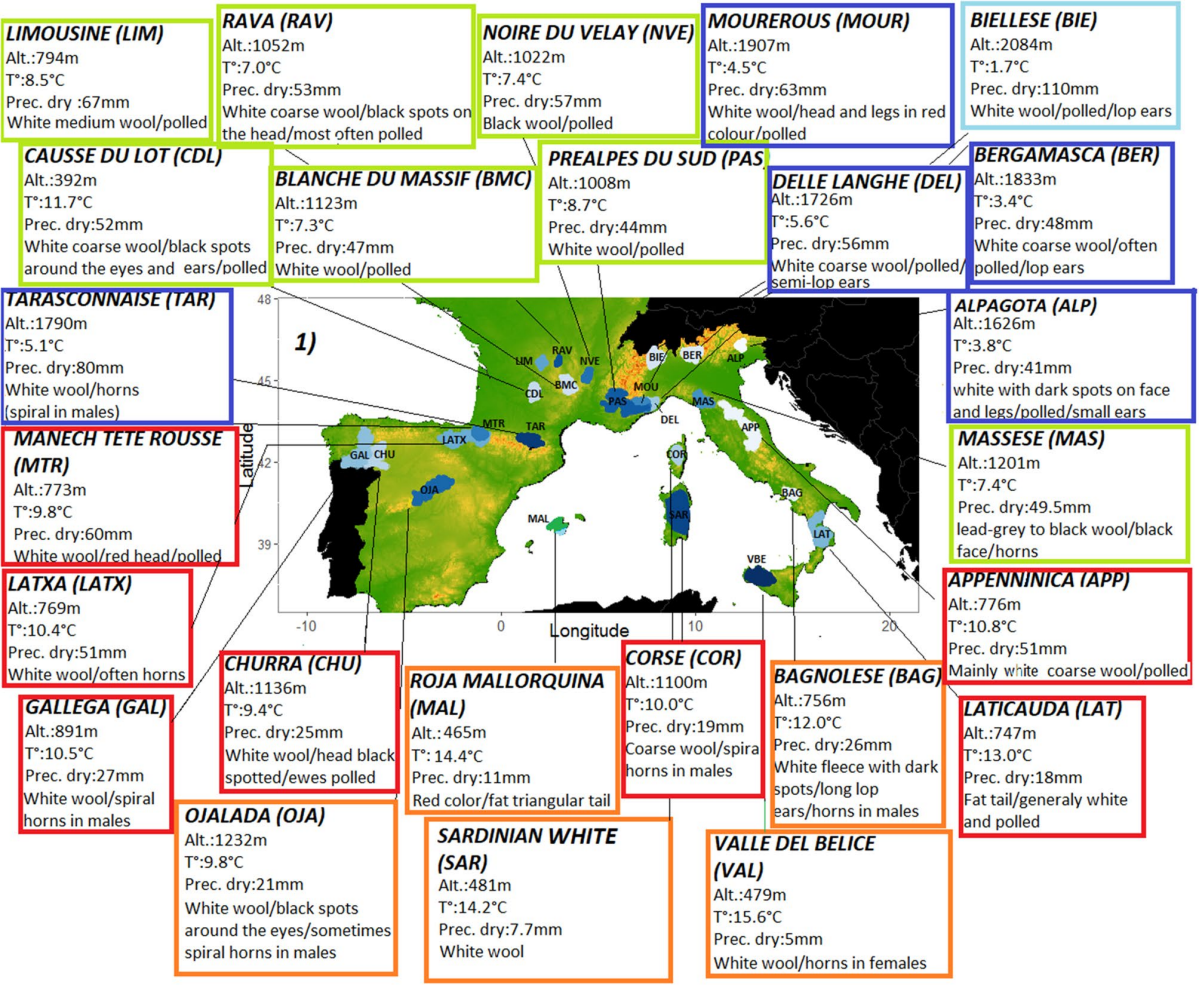
Description of sheep cradles as a function of environmental variables: synthetic descriptions of cradles and some characteristic breed phenotypic traits. The breed labels are color coded according to the clustering ranking defined by the PCA/HCPC analysis shown in Fig. 6.

The dataset includes 17 goat breeds and 25 sheep breeds, selected on the basis of historical and anthropological documentation (Table 1, see Supplementary Table 1 for the initial list of 83 breeds and “Material and methods” section for an explanation of the choice of breeds for analysis). Figures 1 and 2 show the geographical cradles of the different breeds and provide details on the phenotypes of the animals and the environmental conditions at the cradle level.

**Table 1:** Description of datasets.

Breed name	Code	Country	Cradle location
**Goat dataset**			
Bionda dell Adamello	BIO (n = 24)	Italy	The Alpine massif
Valdostana	VAL (n = 24)	Italy	The Alpine massif
Orobica	ORO (n = 24)	Italy	The Alpine massif
Val Passiria	VSS (n = 24)	Italy	The Alpine massif
Di Teramo	DIT (n = 24)	Italy	Abruzzo massif
Ciociara Grigia	CCG (n = 19)	Italy	Aurunci Mountains
Garganica	GAR (n = 20)	Italy	The Gargano promontory
Argentata Dell’Etna	ARG (n = 25)	Italy	Mount Etna
Nicastrese	NIC (n = 25)	Italy	The province of Catanzaro
Girgentana	GGT (n = 30)	Italy	The Agrigento coast in Sicily
Corse	CRS (n = 29)	France	Corsican massifs
Provençale	PVC (n = 18)	France	The "Provence of hills"
Pyrénéenne	PYR (n = 25)	France	Pyrenees
Bermeya	BEY (n = 24)	Spain	Asturias
Blanqua De Rasquera	RAS (n = 20)	Spain	Southern Catalonia
Malaguena	MLG (n = 41)	Spain	Malaga
Mallorquina	MAL (n = 20)	Spain	Island of Mallorca
**Sheep dataset**			
Manech Tête Rousse	MTR (n = 25)	France	Basque hillsides
Tarasconnaise	TAR (n = 15)	France	Central Pyrenees
Causse Du Lot	CDL (n = 20)	France	Plateaus of the Lot
Limousine	LIM (n = 18)	France	Millevaches plateau
Rava	RAV (n = 20)	France	The Massif Central
Blanche Du Massif	BMC (n = 20)	France	The Massif Central
Noire Du Velay	NVE (n = 19)	France	The Massif Central
Préalpes Du Sud	PAS (n = 17)	France	The Southern Alps
Mourerous	MOUR (n = 16)	France	The Maritime Alps
Corse	COR (n = 16)	France	The Corsican massifs
Delle Langhe	DEL (n = 24)	Italy	The Alpine massif
Biellese	BIE (n = 21)	Italy	The Alpine massif
Bergamasca	BER (n = 24)	Italy	The Alpine massif
Alpagota	ALP (n = 24)	Italy	The Alpine massif
Massese	MAS (n = 24)	Italy	The Alpi Apuane mountains
Appenninica	APP (n = 24)	Italy	The Apennine mountains
Laticauda	LAT (n = 24)	Italy	Campania and Calabria
Bagnolese	BAG (n = 23)	Italy	Campania
Valle Del Bellice	VAL (n = 24)	Italy	Sicily
Sardinian White	SAR (n = 24)	Italy	Sardinia
Latxa	LATX (n = 24)	Spain	Pyrenees
Gallega	GAL (n = 27)	Spain	Gallicia
Churra	CHU (n = 30)	Spain	Castile and León
Ojalada	OJA (n = 24)	Spain	The province of Soria
Roja Mallorquina	(MAL, n = 28)	Spain	Island of Mallorca

Breeds, number of individuals, cradle location.

n: number of individuals.

For 15 of the 42 selected sheep and goat breeds considered, we found documented evidence targeting ancestral populations at their origin, dating back several millennia (see Supplementary Table 2 for historical references). The remaining breeds have a history of at least several centuries, with the exception of 4 Italian goat breeds (DIT, CCG, GAR, ARG) and 3 Italian sheep breeds (DEL, BIE, VAL), which were recognized about a hundred years ago under their current names, but which have an ancient history characterized by mixtures of populations native to the territories considered. Seventeen breeds were classified as “critical” or “endangered” according to FAO^20^. Considering jointly the goat and sheep breeds, 30 breeds have been described as showing adaptations to stressful environments and 29 are, or were, transhumant. For most transhumant breeds the transhumance is, or was, vertical, so we included their summer mountain pasture in the geographical definition of the cradle (see Supplementary Table 2).

The Admixture analysis is detailed in Supplementary text 1. For both goats and sheep (Figure 3 in the Supplementary text 1), the cross-entropy curve decreases as K increases, which is characteristic of a distance isolation pattern^21^, moreover, the Mantel tests postulated distance isolation patterns (*p* value = 0.049 for goats and 0.001 for sheep). In this case, to correct for the limited genetic structure, we used K = 1 and K = 2, in the LFMM analyses, as recommended by Dalongeville et al.^22^, De Kort et al.^23^ and Capblancq et al.^24^.

#### Comparison of approaches

**Figure 3:**
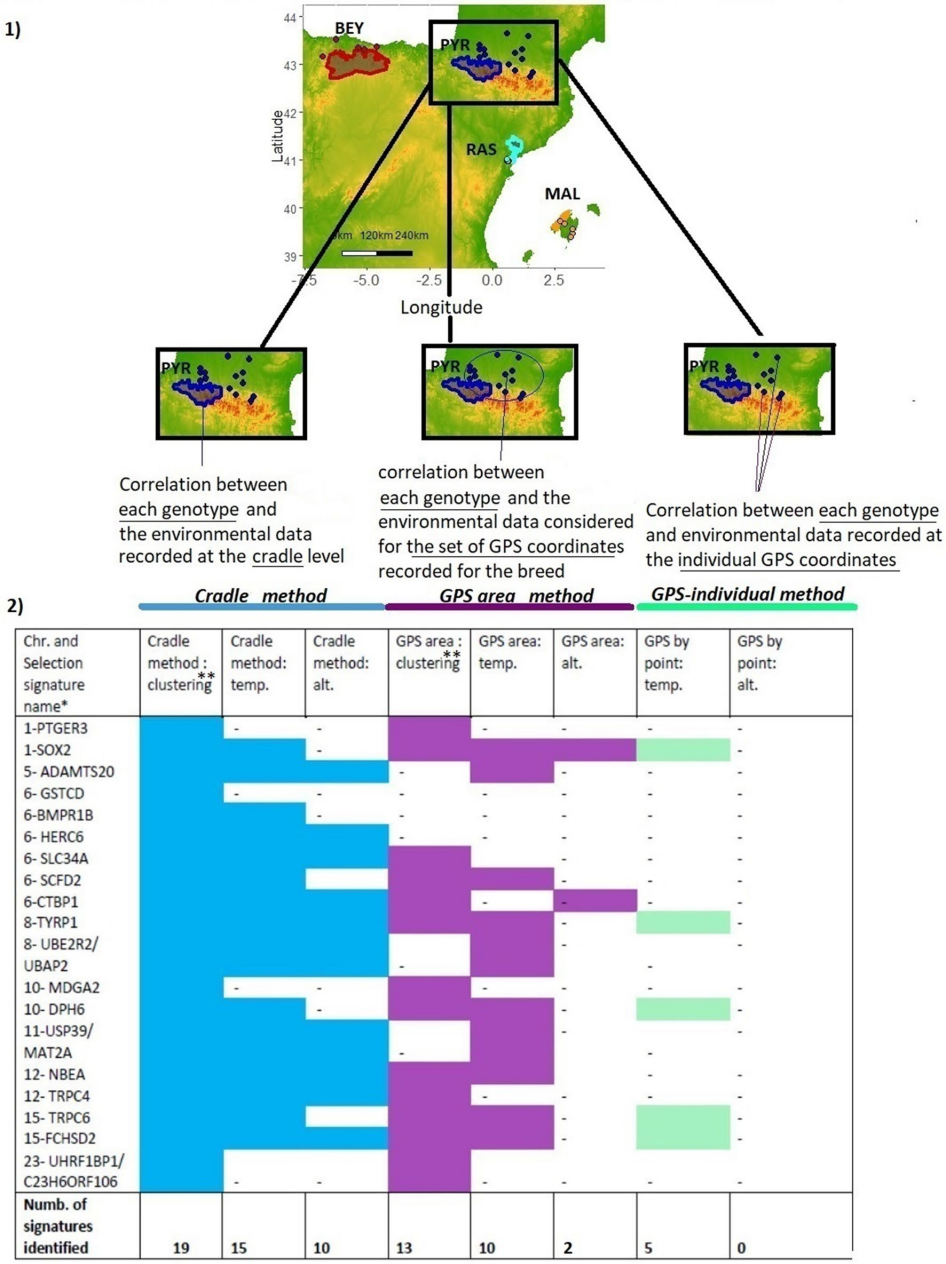
Experimental test of the method. The signature search was done via LFMM. The environment was characterized either at the level of the GPS coordinates (individual GPS method) or considering all the GPS points recorded for a given breed (GPS area method), or at the level of the cradle (cradle method). The environmental variables Altitude and Mean Annual Temperature were considered individually. Chr.: chromosome number. *Selection signature are named according to the gene(s) most likely targeted by the selection process, taking into account the distance of the SNPs from the genes, the associated *p* values and the number of SNPs near or in the gene(s); it is possible to know in detail the genes associated with each selection signature (see Supplementary Table 3). **Clustering: the environment was characterized considering all the variables at the same time and according to the PCA/HCPC process.

We performed the LFMM analyses on the goat dataset using three approaches for characterising the environment of each breed (see “Identification and analysis of genomic selection signatures” and “Experimental test of the method” sections in “Material and methods” section and Fig. 3). The poor results obtained with the Annual Mean Precipitation are not shown (i.e., the different methods could only reveal two signatures each, with NBEA identified in all cases). Notably, for the Altitude, the individual GPS method did not detect any signatures; the GPS area method identified only two signatures, whereas 10 signatures were recorded with the cradle method. For the Mean Annual Temperature, five signatures were observed with the individual GPS method, 10 with the GPS area method, and 15 by the cradle method. The process of clustering the environments allowed the detection of 13 signatures via the GPS areas and 19 (maximum value) via the cradles (see details in Supplementary Figures 3 and 4).

#### Goat dataset: track for selection signatures

From the 37 signatures, 21 were identified by PCAdap, 19 by LFMM; 3 signatures were supported by both methods (Table 2). Seven signatures were observed on chromosome 6, and four on chromosome 12. Genes associated with these signatures have been documented in the literature (Supplementary Table 3). Of the 21 signatures highlighted by PCAdapt, 15 were found to be involved in adaptive processes and of the 19 signatures highlighted by LFMM, 15 were linked to adaptive processes according to the literature.

**Table 2:**
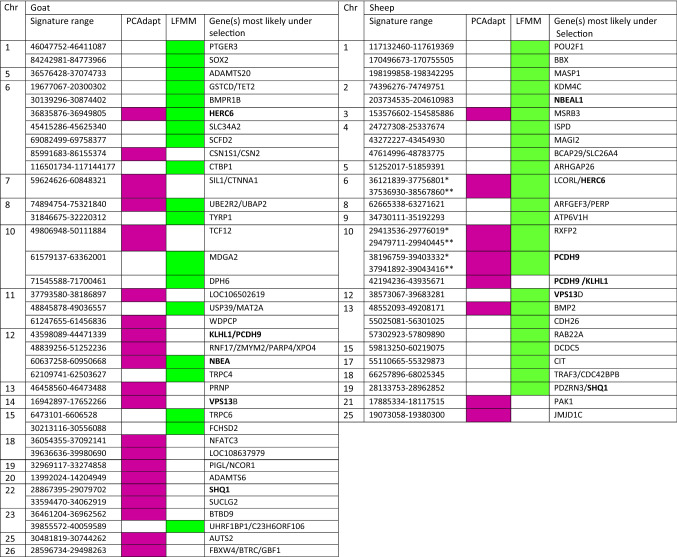
Selection signatures identified in goats and sheep by LFMM and PCAdapt using the cradle method.

Each signature was named according to the gene(s) most likely targeted by the selection process, taking into account the distance of the SNPs from the genes, the associated *p* values and the number of SNPs near or in the gene(s). All annotated genes associated with each selection signature are highlighted in Supplementary Tables 3 and 4.

*PCAdapt signature; **LFMM signature; Chr.: chromosome number, in bold genes identified in both datasets.

**Figure 4:**
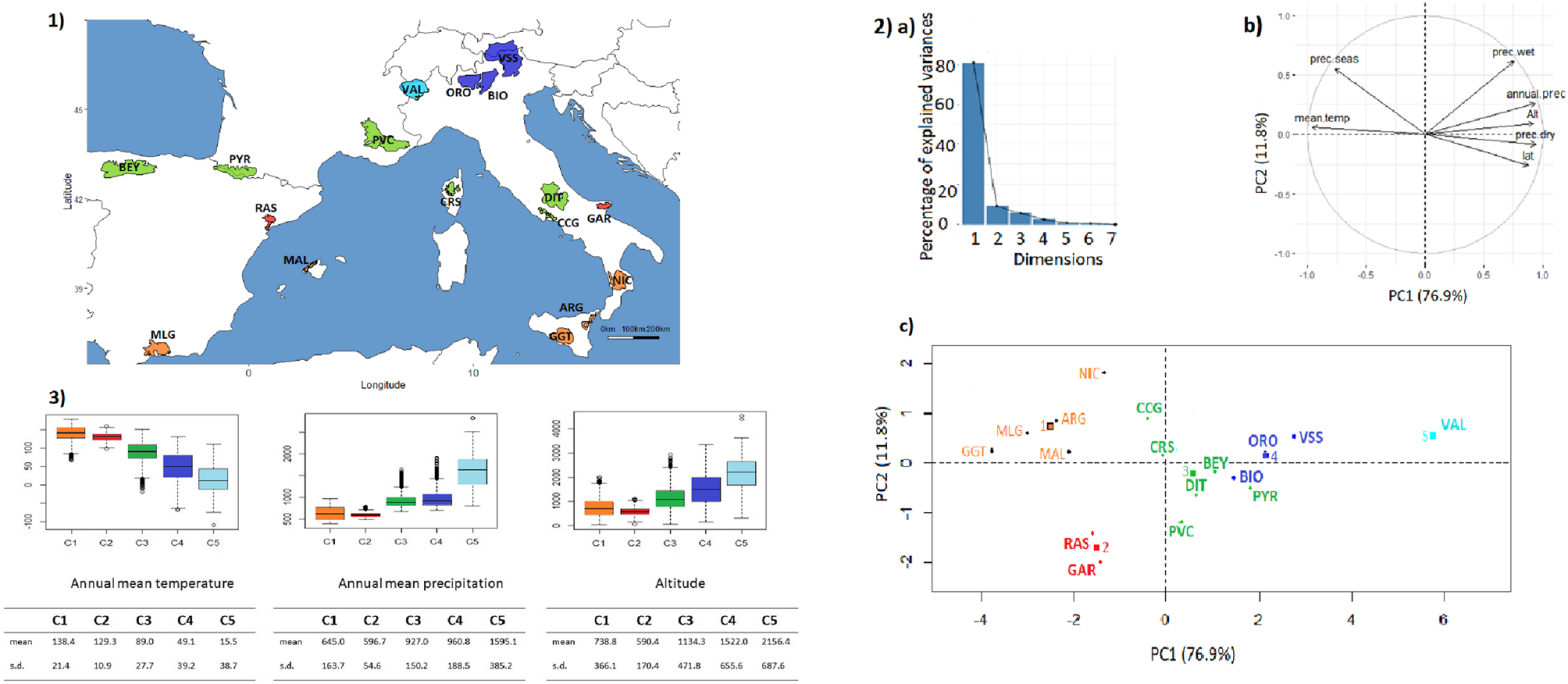
Goat cradles analyses as a function of environmental variables. (**1**) Mapping display of the cradles coloured according to the result of the clustering. (**2**) HCPC analysis. (a) Scree plot. (b) PCA correlation circle. (c) PCA score plot of goat cradles. Colours represent the main clusters obtained by HCPC (K = 5). PC 1 and PC 2 represent respectively principal components 1 and 2; numbers in brackets show the variance explained by each PC. (**3**) Boxplot representation for the variables Mean Annual Temperature, Mean Annual Precipitation and Altitude for the different HCPC groups and associated numerical values. Alt.: Altitude in meters, T°: Temperature in °C × 10, prec.: precipitation in millimeters, min.: minimal, max.: maximal, s.d.: standard deviation.

The LFMM analysis was based on the PCA (Principal Component Analysis)/HCPC (Hierarchical Clustering on Principal Components) ranking in which the cradles were clustered according to the similarity of their environments (Fig. 4). The PCA analysis was largely driven by the first component (76.9% of variance explained by PC1). The cradles appeared to be distributed along a South–North axis, following a decreasing temperature and aridity gradient towards the negative values of PC1, and an increasing altitudinal gradient towards the positive ones. HCPC clustering, indicating K = 5 as the optimal number of clusters, was as follows: the first group (1) clustered breeds from southern Italy, Sicily, southern Spain and the island of Majorca, i.e. regions with high annual mean temperatures and very low rainfall during the dry season. Group (2) clustered the Spanish (Blanca de Rasquera, RAS) and Italian (Garganica, GAR) breeds, raised under low rainfall also during the wet season. The third group (3) clustered Spanish, French and Italian goat breeds, in an intermediate position with regard to the aridity gradient during the dry season. Groups (4) and (5) corresponded to Italian breeds in regions of high altitude, low average temperatures and high precipitation (Fig. 4). PCA/HCPC processed with environmental data recorded at the GPS area level (see Supplementary Figure 4) also showed five clusters that followed a temperature and precipitation gradient, but without an altitude gradient (Supplementary Figure 4.3), resulting in a different ranking clustering than the one obtained by the cradle method (Fig. 4) and a lower number of signatures highlighted by LFMM (Fig. 3).

**Figure 5:**
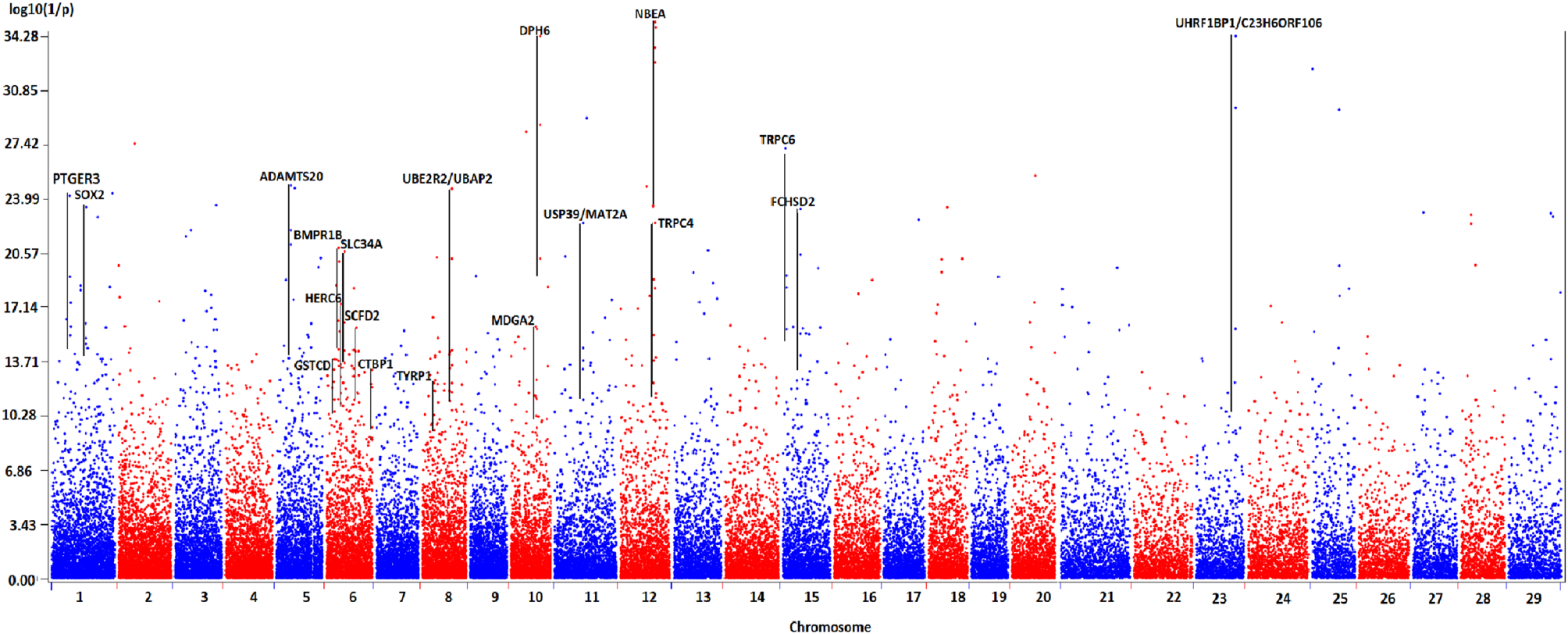
Genome scan for selection signatures in local goat breeds obtained by the LFMM approach and via the cradle method. Each signature was named according to the gene(s) most likely targeted by the selection process, taking into account the distance of the SNPs from the genes, the associated *p* values and the number of SNPs near or in the gene(s).

The LFMM analysis, based on the cluster ranking obtained by the cradle method (Fig. 5), showed several strong signals near genes that have been implicated in adaptation: SOX2, ADAMTS20, UBE2R2/UBAP2, DPH6, NBEA, TRPC4, TRPC6, and UHRF1BP1/C23H6ORF106. Strong signals detected by PCAdapt analysis and also found in the literature as implicated in adaptive processes corresponded to the genes SIL1/CTNNA1, UBE2R2/UBAP2, SHQ1, SUCLG2 as well as a large area on chromosome 12 containing RNF17, ZMYM2, PARP4 and XPO4 and the vast intergenic area between KLH1 and PCDH9. A particularly strong signal was found on chromosome 18 at locus LOC108637979. The detailed results can be viewed in Supplementary Tables 3, 5 and 6.

#### Sheep dataset: track for selection signatures

From the 26 selection signatures, 8 were identified by PCAdapt, 23 by LFMM; 5 signatures were detected by both methods (Table 2). Genes associated with these signatures have been documented in the literature (Supplementary Table 4), all PCAdapt signatures and 18 out of 23 LFMM signatures could be linked to environmental adaptation.

**Figure 6:**
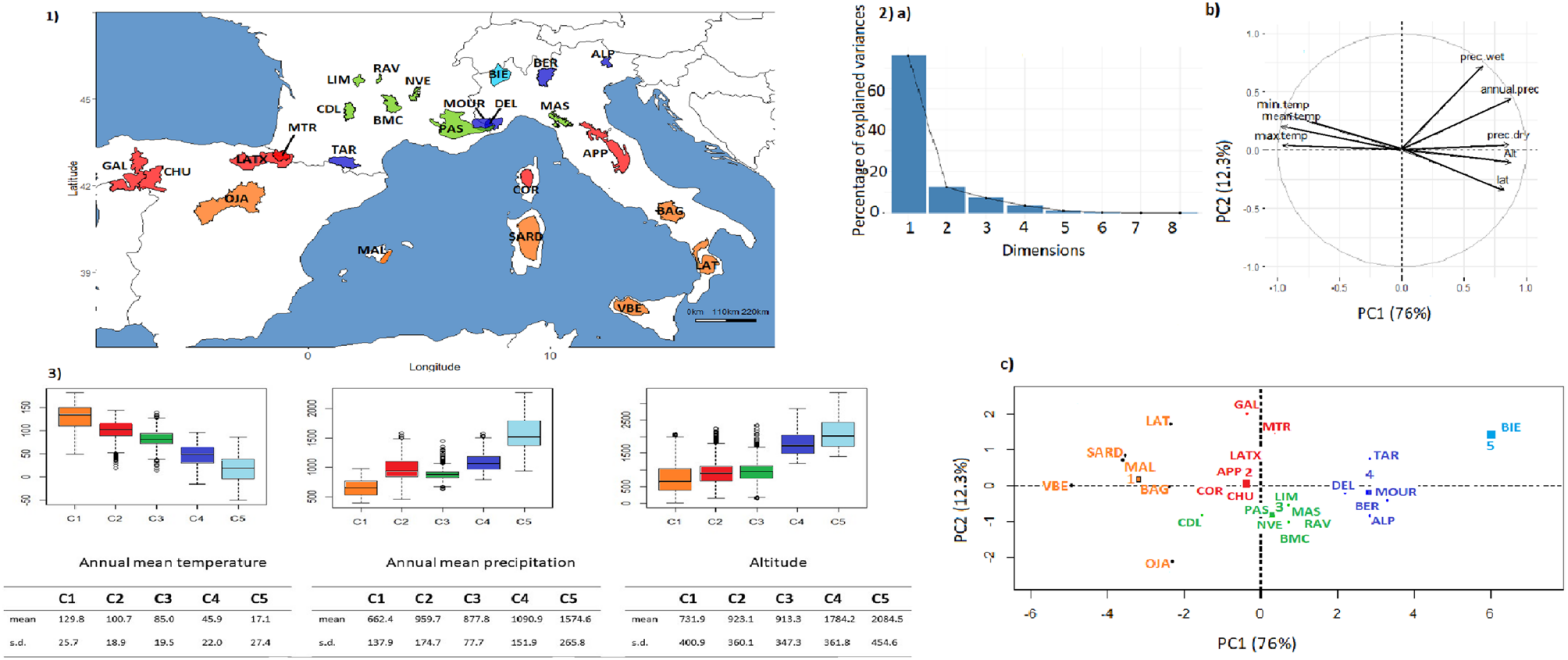
Sheep cradles analyses as a function of environmental variables. (**1**) Mapping display of the cradles coloured according to the result of the clustering. (**2**) HCPC analysis. (a) Scree plot. (b) PCA correlation circle. (c) PCA score plot of goat cradles. Colours represent the main clusters obtained by HCPC (K = 5). PC 1 and PC 2 represent respectively principal components 1 and 2; numbers in brackets show the variance explained by each PC. (**3**) Boxplot representation for the variables Mean Annual Temperature, Mean Annual Precipitation and Altitude for the different HCPC groups and associated numerical values. Alt.: Altitude in meters, T°: Temperature in °C × 10, prec.: precipitation in millimeters, min.: minimal, max.: maximal, s.d.: standard deviation.

**Figure 7:**
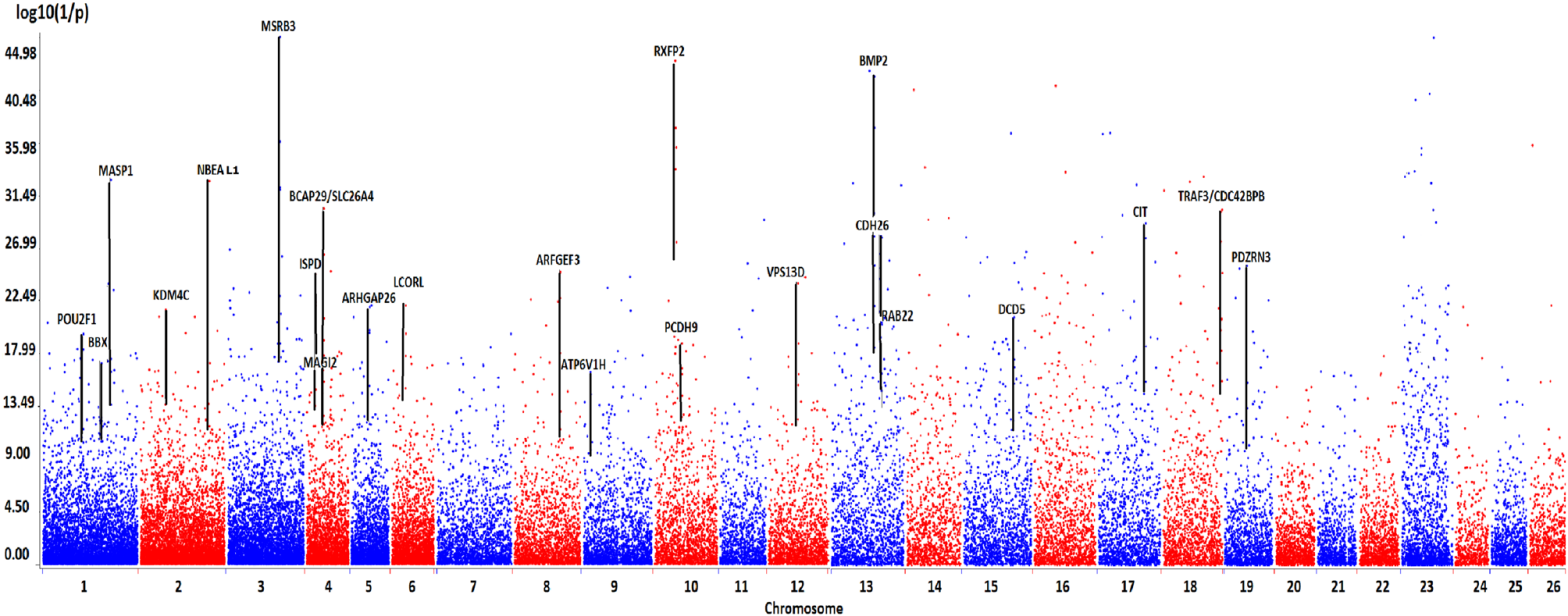
Genome scan for selection signature in local sheep breeds obtained by the LFMM approach and via the cradle method. Each signature was named according to the gene(s) most likely targeted by the selection process, taking into account the distance of the SNPs from the genes, the associated *p* values and the number of SNPs near or in the gene(s).

Figure 6 shows the PCA/HCPC results used for the LFMM analysis. As in Fig. 4 for goats, PC1 accounted for a large part of the variation (76.0%) and showed similar South-North gradients of temperature and aridity (Fig. 6). The optimal K = 5 generated the following clusters: group (1) concerned regions with highest aridity and temperature levels, including the Sicilian, Southern Italian, the island of Majorca and Central Spanish breeds. Groups (2) and (3) were in an intermediate position, in term of dryness and wetness. Group (2) corresponded to regions showing slightly higher average temperatures than group (3) and high levels of precipitation during the wet season (especially for Gallega GAL, Manech Tête Rousse MTR and Latxa LATX), while group (3) included regions of higher altitudes (except for Causse du Lot CDL). Groups (4) and (5), including the Pyrenean and Alpine breeds of the three countries, corresponded to areas of high altitudes and significant precipitation levels (Fig. 6). LFMM on the basis of this cluster ranking obtained by the cradle method (Fig. 7) showed several strong signals near genes that previously have been implicated in environmental adaptation: NBEAL1, MSRB3, RXFP2, BMP2. Several other regions identified by LFMM included genes not previously been implicated in environmental adaptation (Supplementary Table 4).

The strongest PCAdapt signals corresponded to: PAK1 and the KLHL1/PCDH9 intergenic region, both described previously as being involved in environmental adaptation. A broad signal was on chromosome 6, near the genes HERC6, SPP1, LAP3, also previously involved in adaptation. The detailed results can be viewed in Supplementary Tables 4, 5 and 7.

#### Comparison of sheep and goat signatures

The genes NBEA (NBEA in goat and NBEAL1 in sheep), SHQ1 and VPS13 genes (goat VPS13B and sheep VPS13D), the regions containing HERC6 and ABCG2 as well as the KLHL1/PCDH9 intergenic region appeared to be under selection in both goat and sheep (Table 2). Most of the selection signatures (74%), or genes included in the signatures, were found in the literature on mammals and birds as being related to environmental adaptation (Supplementary Tables 3 and 4). Of these, the most noteworthy include the following: 16 goat and 14 sheep selection signatures previously identified in Chinese sheep adapted to extreme environments^9^, four signatures in goats and three in sheep found in Ethiopian sheep kept at high-altitude^7^ and three goat and six sheep signatures detected in Egyptian sheep and goats from arid environments^10^.

## Discussion

This study proposed a new genomic landscape approach for the identification of selection signatures in livestock breeds. The key aspects of this approach are (i) filtering the breeds to be included in the study and (ii) matching the genotypes to the environmental conditions recorded not at the current coordinates of the individuals, but at the level of the areas identified as their cradle of origin.

### Performance of cradle versus GPS method

We targeted French, Spanish and Italian breeds with strong historical links to their territory of origin, in order to build datasets for analysis. For most of the breeds, this link was present for several centuries. The majority of the 63 signatures identified in the current study had not been detected in the initial studies of these datasets^24–30^. A high proportion (74%) of the highlighted selection signatures were previously identified by studies on environmental genetic adaptation in birds and mammals from different parts of the world, providing support for our approach. Further corroboration came from the many signatures identified in our study that have been previously reported in sheep or goats from extreme environments in different parts of the world (^7, 9, 10, 31, 32^, see Supplementary Tables 3 and 4 for a complete list), and also from the several signatures found in both sheep and goats. We applied particularly stringent screens and reinforced our results with a thorough analysis of the literature in order to limit false positives as much as possible. Although our results suggest that the conducted approach can complement classical approaches by providing new results, formal mathematical modeling as well as the identification of candidate mutations and the appropriate functional analyses are required to validate the results.

Results from our approach highlighted a few points. First, the vast majority of small ruminant breeds have been shaped by traditional transhumant breeding practices. However, the individual GPS method will be able to consider summer grazing areas (i) only for breeds and herds always managed according to these practices while transhumance practices are declining and (ii) only if the sampling is carried out during the summer period. Consistent with this, we observed that GPS methods revealed very few signatures with the Altitude variable, whereas it was possible to detect 53% of the total signatures identified with the cradle approach by the consideration of this single variable. Secondly, considering the Mean Annual Temperature variable, (i) the difference between the number of signatures detected by the GPS methods and the cradle approach was clear but less pronounced than for the Altitude variable and (ii) the correlation of genotypes with environmental variables recorded at the level of the area defined by the set of the breed’s GPS coordinates (GPS area method) detected more signatures than considering the environmental variable at individual GPS coordinates (individual GPS method). This suggested that there are benefits of smoothing environmental data at the area level rather than considering it at individual points. Finally, the greatest number of selection signatures was identified by considering all the environmental variables at the same time and using the cradle method including transhumant pasture areas.

Thus, there are several strengths of our novel cradle method. First, taking into account the history of the breeds avoids including breeds that could interfere with the signal (i.e. breeds which do have a strong link with a given environment or whose link has been distorted). Secondly, in industrialized countries, agricultural intensification in recent decades has led to major shifts in practices, particularly the abandonment of transhumance, and also significantly changed the distribution areas of herds. Thus, the environmental characteristics recorded at the current GPS points are different from those of the original cradle (see Supplementary Text 2 for details). Considering the cradle of the breed rather than the current distribution thus links with more accuracy the genetic determinants of adaptation with the environment that has mainly shaped the breed's genome. Thirdly, we combined the PCAdapt algorithm, which is particularly suited to the detection of selection signatures in heterogeneous populations^18^, with LFMM, which identifies the links between genomic and environmental variation. On the basis of the scientific literature we eliminated PCAdapt signatures that appeared to be related to agronomic selection and researched the links established in the literature between other signatures and environmental adaptation. It can be hypothesized that PCAdapt signals not detected by LFMM reflect local adaptation to patchy environmental conditions unrelated to the environmental gradient. The two methods thus provide complementary information. It can be noted that the cradles were organized in both datasets according to a gradient of temperature and altitude, as shown by the PCA analyses; LFMM thus highlighted areas of the genome whose variation was linked to this gradient. The correlation between temperature/humidity variables and altitude is a classic issue in ecology that can be addressed by including microclimatic variables^33^ and/or considering datasets designed in such a way as to allow the evaluation of temperature variations while altitude remains unchanged, or vice versa. These considerations provide an opening for further research that would improve our ability to link signatures more specifically to a specific factor.

It should be noted that the breeds, considered today, come from ancestral populations whose histories are largely unknown. Indeed, the various traces and historical documents indicating the existence of breeds on territories for several centuries, or even millennia, refer to the ancestral populations from which they originated. Hence, selection signatures identified may predate the formation of the breed or alternatively, reflect rapid recent changes. In addition, environmental features of a cradle may not be unique and may also have changed over time. The environmental characterization was inferred from climatic data recorded during the twentieth century. This constitutes a bias, which in the context of current climate change, must be carefully considered. We expect that our work will contribute to the synthesis of landscape genomics studies (e.g.^34^) by focusing on the particularities of livestock at the interface of the natural and anthropized environment.

### Selection signatures identified in this study

The highlighted selection signatures can be categorized according to physiology and phenotypes. In Supplementary Text 3, we discussed the genes that seem to be involved (i) in lipid storage, (ii) in seasonal patterns and circadian behaviors, (iii) in coat colour and horns, (iv) in hypoxia and/or heat stress response, (v) in immunity, (vi) in lung function, and (vii) in neuronal function. We also discussed selection signatures that seemed difficult to classify because of their length and the diversity of the genes within them. Here we only focus on the most robust signals, considering both the *p* values and the number of SNPs involved in the selection signatures, and for which other studies have previously shown a link between the genes involved in the signatures and environmental adaptation.

#### Goat selection signatures

We identified SUCLG2 in goat by the PCAdapt approach. It was found under selection in sheep in arid Egyptian environments^10^ and in the Chinese desert^9^, but also in yaks adapted to extreme altitudes^35^. The gene is involved in the propanoate metabolic pathway and was significantly associated with growth in pigs^36^. Moreover, the latter study suggested that SUCLG2 plays a key role in the regulation of POU1F1, which is well known to be involved in growth function, and belongs to the same family as POU2F1, detected in sheep by our study. The study by Schmidt et al.^37^ in the blind subterranean mole rat, known to show remarkable tolerance to hypoxia and cancer resistance, also highlighted SUCLG2 and POU1F1 in the liver transcriptome. It was hypothesized that the energy-saving responses triggered in hepatic metabolic pathways may be crucial adaptations to low oxygen levels. Finally, the study of Tian et al.^38^, assessing energy metabolism-related genes in hypoxia-tolerant mammals, identified SUCLG2 as candidate gene of interest in the liver of cetaceans.

SOX2 and DPH6 were found correlated with the environmental gradient in goats by LFMM. In mice, SOX2 is expressed in adult SCN neurons and positively regulates transcription of the core clock gene, Period2, implicated in behavioral rhythms linked to environmental light cycles^39^. A link between SOX2 and cold adaptation has been found in marmots^40^. Interestingly, BBX, another Sox protein that belongs to the HMG box superfamily of DNA-binding proteins, was also identified in sheep in our study. DPH6 belongs to the circadian rhythm-related GO categories. Moreover it was found under selection in northern European cattle^41^ and yaks^42^, and associated with plateau conditions in Chinese sheep^9^.

It is interesting to note that the TRPC4 gene, on chromosome12, and the TRPC6 gene, on chromosome 15, are among the strongest signatures highlighted by the LFMM approach. Transient receptor potential channel (TRPC) proteins have been characterized as molecular substrates mediating receptor-activated cation influx. TRPC4 and TRPC6, in particular, have been shown to strongly contribute to synaptic information transfer in neuronal dendrites via the Ca^2+^-dependent release of neurotransmitter^43^. The increase in dendritic γ-aminobutyric acid (GABA) release from thalamic interneurons appears critically dependent on these TRP proteins^44^. Hence, these genes may mediate hot and cold sensation and affect endothelial-dependent regulation of vascular tone^45^. Moreover, there is strong evidence for an important function of TRPC6 in pulmonary vasculature^46^ and a functional role in hypoxia induction, potentially via the metabolism regulation of HIF-1α^31, 32, 47, 48^. In addition, TRPC6 was associated with high altitude adaptation in Tibetan highlanders^49^ and TRPC4 was implicated in body temperature regulation in cattle^50^.

#### Sheep selection signatures

Both PCAdapt and LFMM approaches identified the gene MSRB3. This gene has been subject to high selection pressure in sheep^51^ and has been suggested as a functional candidate for associations with ear morphology in numerous studies. It has been reported in large-eared sheep^52^. Its link with ears was also highlighted in pigs^53, 54^ and dogs^55^. Our study showed a correlation between SNPs near this gene and the environmental gradient. Interestingly, a recent study^56^ associated genetic variation in this gene and fat deposition in sheep. Furthermore, Webster et al.^55^ revealed that this gene was in genetic linkage with variants in HMGA2, a neighbouring gene, which influences body mass in dogs. Moreover, HMGA2 is known to be involved in adipose tissue, development and obesity in mouse^57^. This selection signature, for which the role of MSRB3 and HGMA2 is not yet elucidated, appears to be of central importance in terms of adaptation, because it was identified under selection in highland Ethiopian sheep^7^, in Tibetan sheep^8^, in Tibetan Yaks^35^ and in Tibetan dogs^58^.

Like MSRB3, both methods (PCAdapt and LFMM) implicated BMP2, which initiates osteoblast and adipocyte differentiation. It has previously been found in association with dry conditions in Chinese^9^ and Egyptian sheep^10^. It was also suggested to be under selection at high altitude, in Tibetan sheep^8^ and in yaks^35^. Adaptive mechanisms governed by BMP2 could be linked to lipid storage capacities, particularly at the level of the tail^56, 59, 60^.

Finally, RXFP2, well known to be under strong selection due to its role in horn development^26, 51, 61, 62^, was identified by both PCAdapt and LFMM approaches (see details in Supplementary Text 3).

#### Selection signatures highlighted in both species

An interesting result concerns the area between the PCDH9 and KLHL1 genes that was identified as a selection signature in both sheep and goats. This region appears to be a genetic desert that extends over a syntenic segment conserved on bovine chromosome 12, goat chromosome 12 and sheep chromosome 10. Kim et al.^10^ also found this genomic region to show evidence of selection in both sheep and goats that are adapted to arid environments, and suggested a major role for this region. PCDH9 has been found to be implicated in autism disorders^63^. Protocadherins are thought to be implicated in various aspects of neuronal development and functions. PCDH9 in particular is involved in synaptic cell adhesion^64, 65^. KLHL1 was found related to neuron motion and neuromuscular process^66^ and it may play a role in organizing the actin cytoskeleton of the brain cells.

Another selection signature common to both species and most interesting is the neurobeachin signal (NBEA in goat, and NBEAL1 in sheep). In the present study, LFMM revealed a correlation of NBEA and the environmental gradient, in both goats and sheep; in goat it was also identified by PCAdapt. NBEA was previously reported to be associated with high altitude in Ethiopian sheep^7^, Chinese sheep^9^, cattle^67^, and yaks^35^. Furthermore, it was previously associated with body temperature regulation in cattle^50^ and was suggested to be under selection in Ugandan and Moroccan goats^68, 69^. NBEA is a brain specific A-kinase anchor protein (AKAP), which is required for synaptic surface expression of glutamate and GABA receptors. Neurons lacking the BEACH (beige-Chediak/Higashi) domain protein Neurobeachin (NBEA) show strongly reduced synaptic responses caused by a reduction in surface levels of glutamate and GABAA receptors. Hence, NBEA plays an essential role in thermal adaptation through the regulation of synaptic transmission^70, 71^.

Interestingly, Alberto et al.^72^ identified NBEA, and also, HERC6 and SLC34A2, all detected in the current study, as involved in selective sweeps that differentiate domestic from wild sheep and goat populations, possibly indicating predomestic selection on these genomic areas.

Finally, the signature near NBEAL1 is bordered in sheep by BMPR2, for which mutations have been associated with high Altitude Pulmonary Hypertension (APH) in Kyrgyz Highlanders^73^ and in cattle^74^. Moreover, this gene was found to be associated with desert and plateau conditions in Chinese sheep^9^. BMP2, which belongs to the TGF-β superfamily and is able to activate BMPR2, was observed as a major signal in sheep in this study (see above). Hypoxia may act on BMPR2 activity^75^ and on BMP2 signaling in the pulmonary vasculature^76^.

## Conclusion

Our study provides an approach for integrating ecological, historical and cultural approaches in the search for selection signatures in small ruminants, which as domestic animals are at the very interface of natural and anthropized environments. One of the key step is the characterization of the environment at the cradle level, thus targeting the geographic area, including transhumant summer pastures, where the breed has evolved by traditional practices. Local breeds are derived from populations selected for centuries and sometimes millennia and have thus developed long-standing links between genome and environment. In current times, local breeds are increasingly neglected and have become endangered, or in some cases have even disappeared. For those that remain, traditional practices, including the transhumance that has been one of the main driving forces in their development, are being abandoned^16, 17^. This study emphasizes that local breeds are invaluable resources of identifying environmental adaptation in the context of climate changes.

## Material and methods

### Methodology

A summary diagram of the main steps of the proposed approach is shown in Supplementary Figure 1.

#### Selection of breeds

The aim was to keep breeds with the highest probability of having retained strong adaptations to the local environment, i.e. breeds least affected by the disruptions inherent to the intensification of agricultural practices. For the study, we have targeted the Mediterranean arc in France, Italy and Spain. This region has the advantage of harboring closely related sheep and goat breeds despite large environmental contrasts^11–13^. We initially, considered 32 breeds of goats and 51 breeds of sheep (Supplementary Table 1). Subsequently, we selected 17 goat breeds and 25 sheep breeds (Figs. 1 and 2, and Table 1) on the basis of historical and anthropological documentation and according to the following criteria: (i) the breeds are local (i.e. cosmopolitan breeds were excluded); (ii) the breeds have featured in the local tradition for at least one century; (iii) any recorded introgression of exotic breeds was limited and occurred more than 100 years ago. If available, recent evaluations of admixture were also used to select breeds to include (see^25, 26–29, 77^). Outline descriptions of the selected breeds are in Supplementary Table 2.

#### Geographic definition of cradle of origin

We determined cradles of origin on the basis of historical and anthropological documentation. Particular attention has been paid to transhumance practices (see Figs. 1 and 2 and Supplementary Table 2 for details). We used ArcGIS^78^ to map the cradles in such a way as to include areas of summer pastures when so-called vertical transhumance was recorded. The horizontal transhumance, involving movements covering long distances without significant altitude differences^79^, cannot be directly taken into account because a large horizontal range would complicate the definition of the cradles.

#### Environmental characterization

Bioclimatic data were obtained from the WorldClim database (v.1.4^80^) covering the period from 1960 to 1990, with a spatial resolution of 30 arc-seconds in the WGS84 datum. We considered the bioclimatic variables most relevant to highlight the major contrasts in temperature and humidity between the cradles, i.e.: BIO_1_ = Annual Mean Temperature, BIO_5_ = Maximum Temperature of Warmest Month, BIO_6_ = Minimum Temperature of Coldest Month, BIO_12_ = Annual Precipitation, BIO_13_ = Precipitation of Wettest Month, BIO_14_ = Precipitation of Driest Month, BIO_15_ = Precipitation Seasonality (Coefficient of Variation). Altitude information was collected from the SRTM 90 m Digital Elevation Database (v.4.1)^81^. Finally, latitude was taken as a proxy for luminosity and seasonality. This procedure was performed in R 3.5.2^82^ using the R package RSAGA^83^.

Because our objective was not to estimate allele-environment correlations between each SNP and each environmental variable at a time, but to capture the relation between the genome and the environment considered as a whole, the cradles were clustered according to their climatic profiles, following this procedure:

For each cradle, the environmental variables of 10,000 random location points were extracted, using R packages sp^84, 85^ and rgdal^86^.

In order to identify groups of breeds with relatively similar environmental conditions in their cradle area, principal component analysis (PCA) was performed on the environmental variables. Prior to the analysis, Spearman correlation coefficient identified highly correlated variables (i.e. correlation coefficient r ≥ 0.9), in which case the biologically less relevant variable was removed to facilitate graphical presentation. The PCA analysis was followed by hierarchical clustering on principal components (HCPC), using Euclidian distances and Ward’s method. These multivariate analyses were performed with R software, using the FactoMineR package^87^.

The LFMM^19^ analyses were conducted following the cluster ranking of the cradles obtained through the PCA/HCPC procedure. Indeed, this procedure, allows classifying the cradles by considering their climatic resemblance defined by the set of all the recorded climatic variables. This ranking is then used to establish the link between genome and environment by LFMM, in order to optimize the statistical power of the analysis.

#### Genotyping

For goats, we used the AdaptMap dataset, including breeds genotyped with the Caprine SNP50 BeadChip (available via Dryad: https://doi.org/10.5061/dryad.v8g21pt), which contains genotypes for 53,547 SNPs. For sheep, we merged a dataset of French breeds obtained with the Illumina Ovine HD SNP Beadchip (Zenodo repository https://doi.org/10.5281/zenodo.237116) with a dataset of Italian breeds (^77^, provided by the authors) and a dataset of Spanish breeds (^27^, provided by the authors), obtained with the Illumina Ovine SNP50 BeadChip. SNP data from French breeds were extracted from the 600 K variation using the Ovine SNP50 BeadChip coordinates of SNPs on the OAR v3.1 reference genome assembly using Vcftools^88^. Merging with the SNP50 data resulted in 40,455 genotypes. SNPs and animals were pruned with PLINK v1.07^89^ using the following filtering thresholds: (i) SNP call rate ≤ 97%; (ii) SNP minor allele frequency (MAF) ≤ 1%; (iii) animals displaying ≥ 10% of missing genotypes. After filtration of the merged datasets we retained 50,329 genotypes for 416 goats and 32,168 genotypes for 555 sheep (initial datasets included 420 and 576 individuals, respectively).

#### Identification and analysis of genomic selection signatures

We identified loci that may contribute to local adaptation by two methods: (i) the individual-based multivariate approach PCAdapt^18^, via the pcadapt R package, and (ii) the latent factor mixed models LFMM, as implemented in the R package LEA^90^, for testing genotype-environment association.

PCAdapt scans the genome to detect outliers with respect to the population structure. Unlike population-based approaches, the package can incorporate admixed individuals and does not assume prior knowledge of population structure. As recommended by Luu et al.^18^, the optimal number of principal components (i.e., K, the optimal number of genetic groups) was determined using the graphical PCAdapt function, by varying K from 1 to 35, and following “Cattell’s rule”, i.e., keeping PCs that correspond to eigenvalues to the left of the lower straight line in the screeplot^91^. This led us to choose K = 15 for goats and K = 20 for sheep (see Supplementary Figure 6). Candidate SNPs were identified by calculating the False Discovery Rate (FDR, α = 0.05) of the *p* values associated with Mahalanobis distance estimated by PCAdapt, using the R package q value^92^.

LFMM software assesses associations between genetic variation (response variable) and environmental factors (explanatory variables) using a linear mixed model and controlling for neutral genetic structure, such as population history and isolation-by-distance, with (random) latent factors via an MCMC algorithm. The number of latent factors was chosen in accordance with the Admixture^93^ analyses. We ran LFMM using 1000 sweeps for burn-in and 9000 additional sweeps. Since LFMM uses a stochastic algorithm, ten runs with different seeds were performed. We combined the Z-scores from the ten repetitions and computed the median. We then recalibrated Z-scores by dividing by the inflation factor before computing the *p* values. We then chose significant associations based on FDR (α = 0.05) using the R package qvalue. LFMM was used to test for the correlation between the genetic variations and the environment taken as a whole (i.e. the variables were not considered one by one, except to test the method), but the analysis was performed once, analyzing the correlation between genomic variations and the ranking obtained by the PCA/HPCP clustering of the cradles environments.

For both PCAdapt and LFMM, we applied a stringent screen to identify selection signatures: candidate-selected regions were required to have at least 3 SNPs ≤ 500 kb apart, previously identified based on FDR and showing *p* value ≤ 10^−9^. The window was chosen on the basis of previous evidence that LD in sheep and goats does not persist beyond 500 kb (see Supplementary Figure 2). It should be noted that the size of the window considered is widely used and that the selection criteria retained are among the most stringent (see^53, 54, 94–98^). For each analysis, genes within a region spanning 100 kb upstream and downstream of the candidate selection regions, were annotated in such a way that genes including one or more SNPs identified as selected were specifically studied, but genes bordered by SNPs identified as selected were also considered. The chromosomal regions under selection pressure were inspected using the NCBI Genome Data Viewer (https://www.ncbi.nlm.nih.gov/genome/gdv/), Oar_v3.1 for sheep and ARS1 for goats. Biological functions of the genes identified were inferred from the literature. Studies focusing on environmental adaptation signatures involving the same genes as those identified here were compiled. As top candidate genes for adaptation, we considered genes identified by our study and by at least one other study focusing on environmental adaptation.

### Experimental test of the method

We compared the cradle-based method with the use of the individual GPS coordinates. This comparison was carried out only for the goat dataset, for which the AdaptMap Consortium provided us with the geographical locations for all breeds considered.

For each GPS coordinate, the environmental variables were extracted for the circular region of radius of 5 km centered on the GPS point. We considered the most discriminating variables, Annual Mean Temperature, Annual Mean Precipitation and Altitude, in order to compare the average information obtained by breed from the GPS coordinates with that obtained from the cradles.

The LFMM analysis proceeded as follows (see Fig. 3):

- The individual GPS method: we assessed the LFMM correlation between individual genotypes and the variables Annual Mean Temperature, Annual Mean Precipitation and Altitude, considered separately and extracted for each individual GPS coordinate.
- The GPS area method: we considered environmental data for the set of all individual GPS coordinates recorded for the breed. (i) The LFMM correlation was calculated between each genotype and the average value obtained for the different variables considered separately (Annual Mean Temperature, Annual Mean Precipitation and Altitude). (ii) As an alternative approach, in order to group the different GPS areas according to their similarity, we used all the environmental variables recorded at the set of the breed GPS coordinates and followed the PCA/HCPC process as explained in “Environmental characterization” section. Then we tested LFMM correlation between the genotypes and the resulting cluster ranking.
- The novel cradle method: For each breed, we considered environmental data at the cradle level. The LFMM correlation was tested in two steps, as for the GPS area method.

### Complementary analyses

#### LD decay

To provide an insight into the overall levels of LD in the different breeds, genome-wide pairwise r^2^ values of SNPs separated by a maximum distance of 10 Mb, were calculated with PLINK software.

#### Admixture analyses

To assess population genetic structure we used ADMIXTURE software^99^. Prior to these analyses, SNP pruning was used to select a subset of SNPs with minimal linkage disequilibrium via the—indep option of PLINK with the following parameters: 50 SNPs per window, a shift of five SNPs between windows, and a Variation Inflation Factor threshold of two (corresponding to r^2^ > 0.5). SNPs identified by the study as potentially selected were removed. For K = 2 to K = 25, ten independent runs were performed. The entropy criterion was calculated via the sNMF function implemented in the R package LEA to assess the number of ancestral populations that best explains the genotypic data^100, 101^. We used the program CLUMPAK (^102^, http://clumpak.tau.ac.il) to analyze the multiple independent runs at a single K and visualize the results.

#### Mantel test

Finally, we assessed the correlation between genetic distances and geographic distances using the Mantel test via the mantel.randtest function, implemented in the R adegenet package^103^.

## Supplementary Information

Supplementary Information.

## Data Availability

For goats, we used the AdaptMap dataset, shared on Dryad: https://doi.org/10.5061/dryad.v8g21pt. For sheep, we used the French dataset online at the Zenodo repository https://doi.org/10.5281/zenodo.237116, and the Italian dataset partially available in the Figshare repository via https://doi.org/10.23644/uu.8947346 (for the few missing breeds please contact Elena Ciani: elena.ciani@uniba.it and Johannes A. Lenstra: j.a.lenstra@uu.nl).
